# A spatial and dynamic solution for allocation of COVID-19 vaccines when supply is limited

**DOI:** 10.1038/s43856-021-00023-1

**Published:** 2021-08-19

**Authors:** Wenzhong Shi, Chengzhuo Tong, Anshu Zhang, Zhicheng Shi

**Affiliations:** 1 Smart Cities Research Institute and Department of Land Surveying and Geo-Informatics, The Hong Kong Polytechnic University, Hong Kong, China; 2 Research Institute for Smart Cities, School of Architecture and Urban Planning, Shenzhen University, Shenzhen, China

**Keywords:** Infectious diseases, Public health

## Abstract

**Background:**

Since most of the global population needs to be vaccinated to reduce COVID-19 transmission and mortality, a shortage of COVID-19 vaccine supply is inevitable. We propose a spatial and dynamic vaccine allocation solution to assist in the allocation of limited vaccines to people who need them most.

**Methods:**

We developed a weighted kernel density estimation (WKDE) model to predict daily COVID-19 symptom onset risk in 291 Tertiary Planning Units in Hong Kong from 18 January 2020 to 22 December 2020. Data of 5,409 COVID-19 onset cases were used. We then obtained spatial distributions of accumulated onset risk under three epidemic scenarios, and computed the vaccine demands to form the vaccine allocation plan. We also compared the vaccine demand under different real-time effective reproductive number (R_t_) levels.

**Results:**

The estimated vaccine usages in three epidemiologic scenarios are 30.86% - 45.78% of the Hong Kong population, which is within the total vaccine availability limit. In the sporadic cases or clusters of onset cases scenario, when 6.26% of the total population with travel history to high-risk areas can be vaccinated, the COVID-19 transmission between higher- and lower-risk areas can be reduced. Furthermore, if the current R_t_ is increased to double, the vaccine usages needed will be increased by more than 7%.

**Conclusions:**

The proposed solution can be used to dynamically allocate limited vaccines in different epidemic scenarios, thereby enabling more effective protection. The increased vaccine usages associated with increased R_t_ indicates the necessity to maintain appropriate control measures even with vaccines available.

## Plain language summary

The supply of COVID-19 vaccines is limited in many parts of the world, particularly in low-income countries. To assist in the allocation of limited vaccines, a spatial and dynamic solution was proposed, based on a new model to predict risk of COVID-19 onset in urban communities. A case study in Hong Kong indicated that the estimated vaccine usage (30.86–45.78%) under three epidemiologic scenarios was within the total vaccine availability limit. Vaccine usage would need to be increased by more than 7% if the current rate of viral spread was doubled. The proposed solution has the potential to help countries to allocate limited vaccines spatially and dynamically in different epidemic scenarios, thereby enabling more effective protection.

## Abstract (web summary)

Shi et al. propose a weighted kernel density estimation model to estimate COVID-19 risk across communities in Hong Kong. The authors use this data to evaluate potential COVID-19 vaccine allocation strategies in different epidemic scenarios.

## Introduction

COVID-19 has become a global challenge for human beings^1,2^. As of 5 May 2021, COVID-19 has spread rapidly in 222 countries and regions^3^, and 153,526,293 people have been diagnosed with this disease, with a total of 3,213,701 deaths^3^. Since the outbreak of the COVID-19 pandemic^4^, many countries have successively adopted social distancing and other transmission mitigation measures to reduce the risk of others being infected^5,6^. Most people still lack immunity to the COVID-19 virus (SARS-CoV-2) and are thus still vulnerable to SARS-CoV-2 infection^7–10^.

Therefore, ensuring effective SARS-CoV-2 vaccination against COVID-19 is of the highest priority^11,12^. Such a move could possibly end the COVID-19 pandemic and accelerate the recovery of the global economy^13^.

Currently, 280 candidate vaccines are being developed around the world, of which 96 vaccines, according to WHO data^14^ on 4 May 2021, are at the clinical trial stage. Although over 194 countries and states have received more than 841 million vaccine doses and have already started strategic vaccination distribution^15^, not all countries and states are equally well-equipped with medical resources. More than 700 million doses of vaccine have been given globally, but, it appears that more than 87% of vaccinations are concentrated in high-income and upper-middle-income countries, while low-income countries account for only 0.2%. In high-income countries, on average, close to 1 in 4 people have been vaccinated, whereas in low-income countries the figure is only 1 in 500^16,17^. Disparities were further discovered to exist within the nation. It also found evidence of divides along socio-economic lines^18^. On this basis, the WHO has led the launch of the COVID-19 Vaccines Global Access Facility (COVAX Facility)^19^ to ensure an equitable allocation of vaccines around the world to cover the most vulnerable 20%^20^ of the population of each country participating, in particular the lower-income countries. Currently, 190 counties/regions^21^ have participated in the COVAX Facility. Based on this fact, the Strategic Advisory Group of Experts on Immunisation, World Health Organisation (WHO SAGE) has also been proposed to various countries and regions for reference, a vaccine allocation roadmap^22^ compatible with the COVAX Facility. However, the specific feasibility of the allocation roadmap, especially regarding how to carry out dynamic and reliable spatiotemporal vaccine allocation, is still an important consideration.

The formulation of vaccine allocation plans during an infectious disease pandemic is critical in terms of public health response^23^. During the past few influenza pandemics, some countries have chosen the strategy of allocating available vaccine supplies to each region in proportion to the population of that region^23^. Currently, there are a number of guidelines on how to allocate vaccines fairly and thus protect the rights and interests of various groups of people, including those from the WHO SAGE; Johns Hopkins School of Public Health^24^ (JHSPH); National Academies of Sciences, Engineering, and Medicine^23^ (NASEM), US; Advisory Committee on Immunisation Practices^25^ (ACIP), US; and many other authoritative organisations. The degree of application of these guidelines in specific areas, especially at the urban community scale, is still unknown. Further, COVID-19 shows a typical spatial variation trend which changes dynamically^26,27^. Therefore, knowing how to adjust vaccine allocation spatially and dynamically, to cope with the dynamic changing trend of the COVID-19 pandemic is also essential.

The overall aim is to help low-income countries achieve the following goals by allocating the limited supply of vaccines both spatially and dynamically, but with the following in mind: (i) effectiveness: Care must be taken to ensure vaccines are limited to people in direct need, thereby providing the most effective protection by avoiding further rapid spread, as indicated above; (ii) fairness: To ensure the most vulnerable or susceptible subgroups are considered during each stage of the vaccine allocation; (iii) reasonableness: To assess the impact of vaccination, control measures, and behaviour changes on the spatiotemporal distribution of onset risk to better ensure effective vaccine allocation.

In addition, to the above, the costs of vaccines and cold chain logistics with spatial variation, spatiotemporal factors should be considered in the formulation and implementation of vaccine distribution strategies^28^. Thus, if the supply is limited it is urgent to formulate a spatial and dynamic allocation solution for the COVID-19 vaccine at the urban community scale, and hence better enable the achievement of overall vaccination effectiveness with a limited vaccine supply.

The trend of the symptom onset risk of potential patients can better reflect the risk level of the COVID-19 pandemic^29^ due to the lengthy incubation period^30^ of SARS-CoV-2, and the normally delayed corresponding treatment^31^. Therefore, to spatially and dynamically formulate an effective vaccine allocation plan, appropriate data-driven spatial models need to be adopted to dynamically identify the onset risk level in each region. The weighted kernel density estimation (WKDE) model is one such model^32^ and performs retrospective analysis based on the spatiotemporal information of the cases to infer the date of infection of each onset case^32^. In this way, the risk of infection in a specific location caused by the spatial movement of infected individuals can be predicted. Furthermore, based on the characteristics of human-to-human transmission of COVID-19, dynamic mobility data were introduced into the model to enable reliable and location-specific predictions. This adapted model is referred to as the intercity-scale extended WKDE model^29^. This data-driven spatial model can reduce dependence on theoretical assumptions and parameters, suitable for COVID-19, the mechanism of transmission of which is still under study. Currently, however, there are two areas to be further developed for the extended WKDE model: (a) consideration of vaccination, control measures and behavioural changes in the new normal of the COVID-19 pandemic, and (b) further examination of the effectiveness of utilising finer intraurban community scales, even though the current models are performing well, in general, regarding county/intercity scales. As a result, an urban-community-scale WKDE model has been developed during this study. The aim, when taking into account the vaccination, control measures, and behavioural changes in cities worldwide is to influence the symptom onset risk of COVID-19. The newly developed WKDE model will further include the real-time effective reproduction number (*R*_t_) to both indicate real-time transmissibility and quantify^33^ the effect of vaccination, control measures, and behavioural changes in the model.

**Fig. 1:**
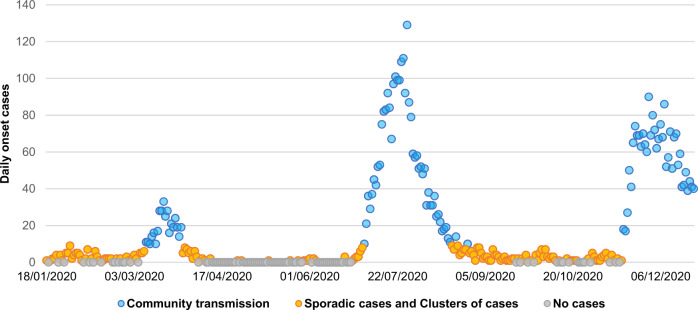
The daily variation in COVID-19 symptom onset cases^35^ in Hong Kong from 18 January 2020 to 22 December 2020 for the three epidemiologic setting scenarios. The blue, orange, and grey dots represent daily onset cases of Hong Kong in the three scenarios of community transmission, sporadic cases and clusters of local onset cases, and no local onset cases, respectively.

In-line with the above, the COVID-19 vaccine allocation plan for Hong Kong (one of the 190 COVAX Facility counties/regions) is to be formulated taking three epidemiologic setting scenarios into account^22^: (i) no local onset cases, (ii) sporadic cases or clusters of local onset cases, and (iii) community transmission. The newly developed urban-community-scale WKDE model is used to predict COVID-19 symptom onset risk in each of the 291 Tertiary Planning Units (TPUs) in Hong Kong, covering a total of 1106 km^2^^34^ (Fig. 1). Five thousand four hundred and nine COVID-19 onset cases^35^ data in Hong Kong from 18 January 2020 to 22 December 2020 is used in this study. The vaccine demand is then estimated for two different situations: (a) before and or (b) during the process of vaccine allocation, under the impact of daily vaccination, or control measures and behavioural changes. Furthermore, the vaccine usage (in proportion to the population vaccinated) needed under different real-time effective reproduction number (*R*_t_) levels, is also be simulated and estimated.

We show that the estimated vaccine usages (30.86–45.78%) in Hong Kong in three epidemiologic scenarios are within the limit of total vaccine availability. The vaccine usages needed will be increased if the real-time effective reproduction number *R*_t_ is increased. Our study demonstrates the feasibility of the proposed solution for allocating limited COVID-19 vaccines spatially and dynamically in different epidemic scenarios.

## Methods

### Data sources

A total of 3977 COVID-19 onset cases with spatiotemporal information in Hong Kong during the period 18 January 2020 to 16 November 2020 were collected from official reports^33^ of the Department of Health of Hong Kong. Excluding the imported onset cases receiving compulsory quarantine and onset cases with unknown location information, 3316 local onset cases were used for this study (Fig. 1). For these 3316 cases, we obtained the available information on dates of onset and reporting as well as the community-level locations, where these patients had stayed prior to diagnosis. According to the transmission categories corresponding to epidemic setting scenarios, the 3316 local onset cases in Hong Kong were classified into three epidemiologic scenarios^22^: (i) no local onset cases (31%), (ii) sporadic and cluster of local onset cases (46%), and (iii) community transmission (23%).

In addition, in order to further simulate the impact of the protection of the population formed by vaccination on the spatiotemporal trend of onset risk, the 2093 local onset cases^35^ with community-scale geographic location from 17 November 2020 to 22 December 2020 were used for this study (Fig. 1). Currently, the main COVID-19 vaccine used in Hong Kong is Pharma/BioNTech Comirnaty COVID-19 mRNA Vaccine-BNT162b2^36^ (also one of the main vaccines allocated by the COVAX facility). It has a vaccine efficacy of 95.0%. Forty-two thousand people can be vaccinated every day^36^. It takes time after vaccination for antibodies to develop in the body and offer protection. Individuals may not be fully protected until 7 days after their second vaccine dose^36^. Hence this study assumes that (i) the interval between the first dose and the second dose is 21 days^36^; (ii) It takes time after vaccination for antibodies to develop in the body and offer protection against COVID. Individuals will be fully protected after 7 days after their second dose of vaccine^36^; (iii) the vaccine allocations in Stage I (very limited vaccine availability accounting for 1–10% of the city’s population) would be in Hong Kong from 16 November, 2020. So accordingly, the Stage II (limited vaccine availability accounting for 11–20% of the city’s population) would start on 4 December 2020, and the Stage III (moderate vaccine availability accounting for 21–50% of the city’s population) would start on 22 December 2020.

**Fig. 2:**
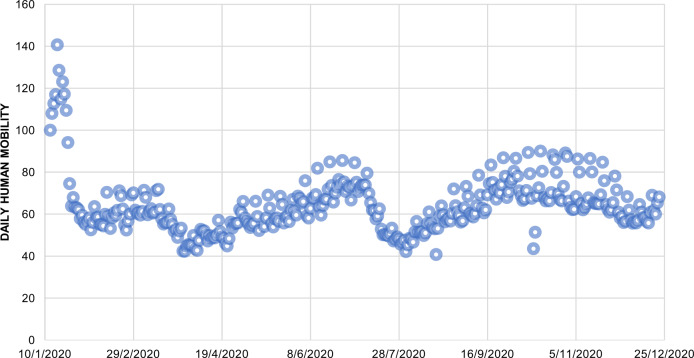
The daily variation in human mobility^38,39^ in Hong Kong from 13 January 2020 to 22 December 2020. The blue dots represent daily community-level human mobility of Hong Kong.

Based on the daily traffic flow data^37^ of 575 closed circuit televisions (CCTV) and traffic detectors covering all Hong Kong strategic routes in Hong Kong, during the same period in 2020 (from 18 January 2020 to 22 December 2020), the traffic flow data within a TPU and between TPUs are used in this research to indicate human mobility within a particular TPU and that from other TPUs to this TPU. These data mainly include two parts: traffic flow data within a particular TPU and that between this TPU and other TPUs. In addition, community-scale daily human mobility data^38,39^ (Fig. 2) during the same period in 2020 (from 18 January 2020 to 22 December 2020), provided by Apple Maps and Google, were used to improve the traditional SIR model to calculate real-time effective reproduction number *R*_t_ for local cases in Hong Kong.

The other 18 categories of the statistics data^40^ in 291 TPUs (Supplementary Table S1), such as the number of medical workers, elderly individuals, school staff, other essential workers outside the health and education sectors, low-income groups, and immigration staff in Hong Kong, were also used in this study.

### The solution to COVID-19 vaccine allocation at the community scale

A solution for how to allocate the COVID-19 vaccine spatially and dynamically in the context of its limited early supply is proposed in this study. The solution is composed of (1) the principles of the allocation, which is based on the WHO SAGE roadmap^22^ for the allocation of COVID-19 vaccines; (2) an urban-community-scale WKDE model for predicting COVID-19 symptom onset risk for different epidemiologic scenarios: (i) no local onset cases, (ii) sporadic cases or clusters of local onset cases, and (iii) community transmission; and (3) an urban-community-scale COVID-19 vaccine allocation strategy for the three scenarios based on these principles and the onset risk prediction results. Furthermore, the impact of the different real-time effective reproduction number levels on vaccine demand and allocation are also evaluated.

#### Principles of vaccine allocation

In this study, the WHO SAGE roadmap^22^ for prioritising the administration of the COVID-19 vaccine in the context of its limited supply is adopted as the overall strategy for COVID-19 vaccine distribution at the urban-community-scale. The reason for this adoption of the WHO SAGE roadmap^22^ as our principle is that we share its goals and can realise these goals quantitatively: (a) reduce the mortality, morbidity, and infection rate brought by the COVID-19 pandemic to cities; (b) prioritise ensuring that key population subgroups have equal access to the vaccine; and (c) reduce the total cost of vaccination and transportation. It should be noted that the assumptions in the WHO SAGE roadmap^22^ are also applicable in this solution.

#### A community-scale WKDE model for predicting the onset risk of COVID-19 symptoms

As a further development of the intercity-scale extended WKDE model^29^, the community-scale WKDE model proposed, includes the following three steps:

- (a) Conducting a retrospective analysis of the historical existence likelihood of the infection in each community location, in which an onset case occurred,
- (b) Making inferences concerning the historical existence of the likelihood of the infection spreading throughout the entire city, and
- (c) Predicting the entire city’s future potential epidemic onset risk on any one given day.

The main difference between the urban-community-scale WKDE model and the extended intercity-scale WKDE model is that at step b) of the model, the historical existence likelihood of the infection occurring at a random location in the entire city has been formulated as follows:

1 $${P}_{{{{{{\rm{Infection}}}}}}}(S,t_{{{{{{\rm{i}}}}}}})\,=\,n{({t}_{{{{{{\rm{i}}}}}}})}^{-1}\mathop{\sum }\limits_{j=1}^{n({t}_{{{{{{\rm{i}}}}}}})}R{{{{{\rm{t}}}}}}(t_{{{{{{\rm{i}}}}}}})M_{{{{{\rm{inter-TPU}}}}}}\,(S,{t}_{{{{{{\rm{i}}}}}}})M_{{{{{\rm{intra-TPU}}}}}}(S,{t}_{{{{{{\rm{i}}}}}}}){P}_{{{{{{\rm{Infection}}}}}}}({L}_{{{{{{\rm{j}}}}}}},{t}_{{{{{{\rm{i}}}}}}}){K}_{{{{{{\rm{h}}}}}}}(S-{L}_{{{{{{\rm{j}}}}}}}),$$

where *P*_Infection_(*S*, *t*_i_) is the probability of any individual, *l* in the city on day *t*_i_, infected with COVID-19 infecting others in random locations, *S, L*_j_ is the *j*th location places where onset cases have occurred^29^. *P*_Infection_(*L*, *t*_i_) denotes the probability that one onset case had been infected on day *t*_i_ in location *L*^29^*. K*_h_(*S* – *L*_j_) denotes a Gaussian kernel^29^.

*R*_t_(*t*_i_) denotes the real-time effective reproductive number for local cases in the city on day *t*_i_^41^_._ The values of *R*_t_(*t*_i_) indicates real-time transmissibility. As there is a delay of several days between an infection and the case report, the traditional framework is unable to provide a real-time estimate of *R*_t_. Leung et al. proposed a new method^41^, whereby human mobility data was used to improve the traditional SIR models to conduct real-time monitoring of community-level transmissibility to obtain real-time estimates of daily *R*_t_ in the following three steps^41^:

i. Estimate the instantaneous reproduction number *R*_t_ of local cases in the city from day *t*_1_ to day *t*_i_.
ii. Correlate the time series (post-average value) of the obtained empirical *R*_t_ estimation with the daily variations of human mobility obtained from different types of data sources in the city.
iii. Select the human mobility values with a high correlation coefficient with *R*_t_ to represent the overall human mobility trend of the city to improve the traditional SIR (susceptible-infected-recovered) epidemic model. That is, on the *t* day, by using the overall human mobility trend as the scaling factor of the SIR model contact matrix, the epidemic curve from day *t*_1_ to day *t*_i_ is further obtained.

However, it needs to be pointed out that in step iii, when the vaccination rate in the city is constantly changing, the removals *R* will increase accordingly, and the susceptibility of *S* will increase. Therefore, the impact of daily variation in the vaccination rate on the epidemic curve from day *t*_1_ to day *t*_i_ is noted.

**Fig. 3:**
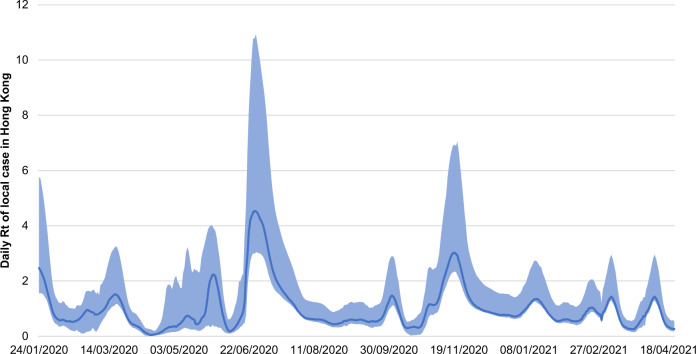
The daily variation in real-time effective reproductive number (*R*_t_(*t*_i_))^41^ for local cases of Hong Kong from 18 January 2020 to 22 December 2021. The blue line and shades represent the mean and 95% uncertainty interval of daily Rt^41^.

Of interest, in this study, the real-time effective reproductive number (*R*_t_(*t*_i_)) for local cases in Hong Kong from 18 January 2020 to 22 December 2020 was estimated (Fig. 3). In Hong Kong, the 95% uncertainty interval of the daily *R*_t_ value was also estimated (Fig. 3). Note that *R*_t_(*t*_i_) after implementation date *t*_239_ in Hong Kong of the current real-time effective reproductive number level is calculated differently in the following three situations: (a) maintaining the current real-time effective reproductive number level, (b) decreasing the current real-time effective reproductive number level, and (c) increasing the current real-time effective reproductive number level. Thus, *R*_t_(*t*_i_) after *t*_239_ is set equal to an actual real-time effective reproductive number, while maintaining the current real-time effective reproductive number level. *R*_t_(*t*_i_) after *t*_239,_ is set equal to half of the real-time effective reproductive number, while decreasing the current real-time effective reproductive number level. *R*_t_(*t*_i_) after *t*_239,_ is set equal to double the value of the real-time effective reproductive number, while increasing the current real-time effective reproductive number level.

*K*_h_(*S* – *L*_j_) denotes a Gaussian kernel^29^:

2 $$K_{{{{{\rm{h}}}}}}({{S}}-L_{{{{{{\rm{j}}}}}}})=\frac{1}{2\pi \sqrt{\det (h)}}\exp (-\frac{1}{2}{(S-L_{j})}^{T}{h}^{-1}(S-L_{{{{{{\rm{j}}}}}}})),$$

where *h* denotes the *d* × *d* bandwidth matrix. By generalising Scott’s rule of thumb, the *d* × *d* bandwidth matrix ${{h}}={n}^{-\frac{2}{d+4}}\mathop{\sum}\limits^{\wedge }$ is chosen here^32,42–44^ with *n* being the total number of onset cases in all 339 days, and $\mathop{\sum}\limits^{\wedge }$ being the covariance matrix of the onset cases sample^29^. For the bivariate distributed onset case samples, the value of d is set as 2^29^. So the bandwidth matrix *h* is equal to ${{{n}}}^{-1/3}\mathop{\sum}\limits^{\wedge }$.

*M*_intra_TPU_(*S*, *t*_i_) denotes a human mobility factor^29^ within a TPU containing location *S* on day *t*_i_, calculated as follows:

3 $${M}_{{{{{{\rm{intra}}}}}}\_{{{{{\rm{TPU}}}}}}}(S,t_{{{{{{\rm{i}}}}}}})={i}^{-1}{\sum }_{k=1}^{i}W_{{{{{{\rm{k}}}}}}},$$

where *W*_k_ denotes the daily traffic flow within the TPU containing location *S* on day *t*_k_ prior to *t*_i_.

*M*_interTPU_(*S*, *t*_i_) denotes a human mobility factor^29^ from other TPUs to the TPU containing location *S*, calculated as follows:

4 $${M}_{{{{{{\rm{inter}}}}}}\_{{{{{\rm{TPU}}}}}}}(S,t_{{{{{{\rm{i}}}}}}})={i}^{-1}{\sum }_{k=1}^{i}V_{{{{{{\rm{k}}}}}}},$$

where *V*_k_ denotes the daily traffic flow from other TPUs to the TPU containing location *S* on day *t*_k_ prior to *t*_i_.

Finally, the daily predicted risk in each location was standardised^29^ to a value between 0 and 1 on a specific date. Different levels of onset risk were set as follows^29^: low onset risk [0–0.2], low-medium onset risk [0.2–0.4], medium onset risk [0.4–0.6], medium-high onset risk [0.6–0.8], and high onset risk [0.8–1]. Similar to the extended intercity-scale WKDE model, the reliability of the predicted COVID-19 onset risk was evaluated by its spatial significance^29^, i.e., the percentage of onset cases on a future date to be predicted that occur in the high onset risk areas^29^ (identified onset hotspot).

Next, based on the daily onset risk prediction results in a specific time period, the illness onset risk prediction was derived for the different epidemiologic scenarios: (i) no local onset cases, (ii) sporadic cases or clusters of local onset cases, and (iii) community transmission. When a new daily onset risk prediction result is added later, the accumulated onset risk in each scenario can be adjusted dynamically.

#### COVID-19 vaccine allocation at the urban community scale

The final allocation of the vaccine at the urban-community-scale is based on the spatial prediction model of COVID-19 onset risk. The allocation strategy was set for three epidemiologic setting scenarios: (i) no local onset cases, (ii) sporadic cases or clusters of local onset cases, and (iii) community transmission. The key subgroups in terms of high infection risk and the predicted spatiotemporal distribution of COVID-19 onset risk in each scenario were comprehensively considered. As a result, spatial and dynamic vaccine allocation at the urban-community-scale was achieved.

In this study, an overall urban-community-scale COVID-19 vaccine allocation plan for the three scenarios (Supplementary Table S2–4) was formulated. For each scenario, a vaccine allocation plan was made according to three possible vaccine supply stages: Stage I: very limited vaccine availability accounting for 1–10% of the city’s population; Stage II: limited vaccine availability accounting for 11–20% of the city’s population; and Stage III: moderate vaccine availability accounting for 21–50% of the city’s population.

Compared with the roadmap of WHO SAGE, based on the characteristics of the proposed urban-community-scale onset risk model, the following improvements have been made to the allocation plan: (a) the consideration of the spatiotemporal distribution of onset risk was highlighted (the susceptible population in high-risk communities was protected first); (b) when considering a high-risk community, other communities with close contacts were also considered; and (c) in Stage III of the sporadic cases or clusters of local onset case scenarios, people travelling to high-risk areas for work were also considered (cross-district staff within communities with high onset risk and with close contacts to communities with high onset risk).

Note that in the application of the Hong Kong solution, given above, the community refers to TPUs. In addition, according to the mortality rate of COVID-19 cases in various age groups in Hong Kong, elderly individuals were defined in the age-based risk group as those over 80 years old. The mortality rates for the different groups were 29.86% (aged 80 years or above), 17.13% (aged 70 years or above), and 11.58% (aged 65 years or above).

### Reporting summary

Further information on research design is available in the Nature Research Reporting Summary linked to this article.

## Results

### The urban-community-scale WKDE model

Based on the urban-community-scale WKDE model developed in this study, the data of onset cases^35^ in 291 TPUs of Hong Kong from 18 January 2020 to 16 November 2020 were applied to obtain the spatiotemporal distribution of the COVID-19 onset risk in its first three waves. To take into account the effective control measures and behavioural changes in Hong Kong, the data of community-scale daily mobility^38,39^ in Hong Kong in the period have been used in the model.

**Fig. 4:**
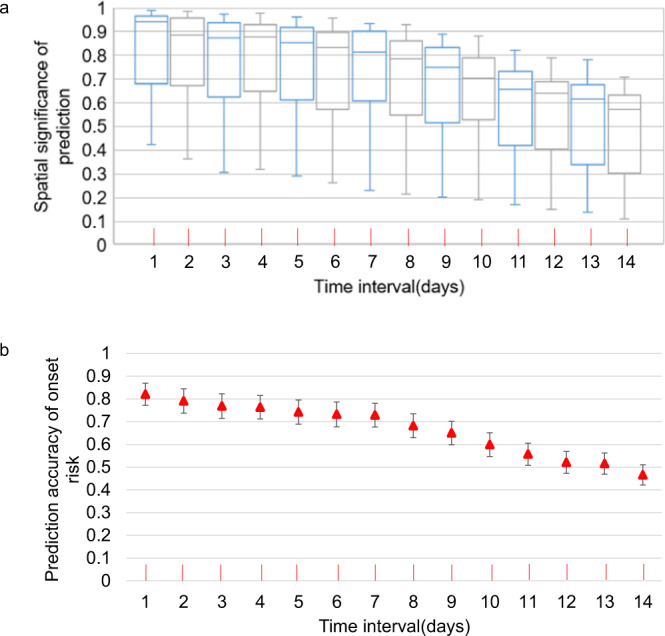
Accuracy^29^ of the predicted risk of COVID-19 symptom onset by urban-community-scale WKDE models, and 95% confidence interval of the prediction accuracy. **a** The prediction accuracy^29^ of urban-community-scale WKDE models. The prediction accuracy is defined as the percentage of the onset cases in the high onset areas^29^. The time interval denotes the time interval from the base date when the prediction is made to the prediction date. The horizontal line in the box, the lower and upper edges of the box denote the median, first, and third quartiles of the prediction accuracy at each time interval^29^. The upwards and downwards lines emanating from the box indicate the maximum and minimum prediction accuracy at each time interval^29^. **b** 95% confidence interval of the mean accuracy of the predicted risk of COVID-19 symptom onset by urban-community-scale WKDE models. *n* = 303 independent samples on each time interval.

Similar to the prediction accuracy analysis^29^ used for the intercity-scale extended WKDE model, the daily prediction accuracy of the COVID-19 symptom onset risk was evaluated by calculating the percentage of onset cases that occurred in community areas, in which the predicted risk of symptom onset was higher than 0.8^29^ (identified as onset hotspots). The prediction accuracy of the community-scale WKDE model was over 70% when predicting the onset risk in the following 7 days (Fig. 4a). The confidence interval of the prediction accuracy was also shown in Fig. 4b.

**Fig. 5:**
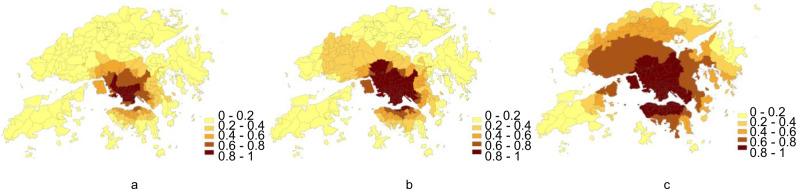
Predicted risk of COVID-19 symptom onset in different epidemic scenarios from 18 January 2020 to 16 November 2020. **a** The accumulated results in the scenario of no local onset cases. The tertiary planning units (TPUs) with higher onset risk values are illustrated in darker colour shading. **b** The accumulated results in the scenario of sporadic cases and clusters of local onset cases. TPUs with higher onset risk values are illustrated in darker colour shading. **c** The accumulated results in the scenario of community transmission. TPUs with higher onset risk values are illustrated in darker colour shading.

The daily spatiotemporal distributions of the COVID-19 symptom onset risk from 18 January 2020 to 16 November 2020 were classified into the different epidemiologic scenarios: (i) no local onset cases (92 days), (ii) sporadic cases and clusters of local onset cases (137 days), and (iii) community transmission (67 days). On the basis of the above prediction results for daily onset risk, the risk in the three scenarios for each TPU (Fig. 5) was calculated by totalling the daily onset risk of the TPUs in each of the three scenario periods (Supplementary Fig. S1). Through the three scenarios, obvious differences were found such as the number of TPUs, in terms of accumulated risk levels. Two epidemiologic setting scenarios are as follows: (i) no local onset cases and (ii) sporadic and cluster of local onset cases—the number of high-risk TPUs was respective 54 and 63. However, in the community transmission scenario, the number of high-risk TPUs reached 134. The spatiotemporal distributions of the onset risk in these three epidemic scenarios were then used as the basis for vaccine allocation in the three scenarios.

### COVID-19 vaccine usage estimates before the vaccines start to be allocated

Based on the proposed vaccine allocation strategy presented in the “Methods” section and the onset risk distribution in the three epidemiologic setting scenarios as follows—(a) no local onset cases, (b) sporadic cases and clusters of local onset cases, and (c) community transmission, the vaccine allocation was calculated for each stage under each scenario. In the no local onset case scenarios (Table 1), in addition to the vaccine usages, which accounted for 9.60% and 6.65% of the total population planned to be used for emergency reserves and travellers abroad in the respective Stages I and II, the total vaccine usages estimated in the three stages was 30.86% of the total population in Hong Kong (2,317,277 people). In addition to the vaccine usages, in the sporadic cases and clusters of local onset case scenario (Table 2), an account of 6.33% of the total population would be used for emergency reserves in Stage I. The further vaccine usages suggested for Stages I, II, and III, were estimated to be 3.67%, 10.74%, and 25.30% of the total population, and hence accounting for 39.71% of the total population in Hong Kong (2,981,824 people). In the community transmission scenario (Table 3), the vaccine usages in Stages I, II, and III were estimated to include: 5.61%, 14.21%, and 25.96% of the total population, respectively, thus accounting for 45.78% of the total population of Hong Kong (3,437,620 people).

**Table 1:** COVID-19 vaccine usage in the no local onset cases scenario.

Vaccine supply scenario	Priority groups	Vaccine usage in each substage (%)	Vaccine usage in each stage	Total vaccine usage (%)
Stage I (Very limited vaccine availability accounting for 1–10% of the city’s population)	Stage Ia: Front-line medical workers in communities with high onset risk and with close contacts to communities with high onset risk	0.28	0.40% (With 9.60% for essential travellers at risk + emergency reserve in Stage I)	0.40
	Stage Ib: Border protection staff and workers for outbreak management	0.12		
	Stage Ic: Essential travellers facing risk of infection outside Hong Kong Stage Id: Emergency reserve utilisation for focused outbreak response	9.60		
Stage II (Limited vaccine availability accounting for 11–20% of the city’s population)	Stage IIa: Front-line medical workers in the remaining low-risk to medium-high-risk communities	0.69	3.35% (With 6.65% for remaining travellers at risk + emergency reserve in Stage II)	3.75
	Stage IIb: Elderly individuals aged 80 years or above with medium-high or higher onset risk and communities with close contacts to communities with medium-high or higher onset risk	2.66		
	Stage IIc: Remaining travellers facing risk of infection outside Hong Kong Stage IId: Emergency reserve of vaccines utilisation for outbreak mitigation	6.65		
Stage III (Moderate vaccine availability accounting for 21–50% of the city’s population)	Stage IIIa: Elderly individuals aged 80 years or above in communities with low to medium onset risk	1.98	27.11	30.86
	Stage IIIb: School staff	1.19		
	Stage IIIc: Other essential workers outside the health and education sectors	23.94

**Table 2:** COVID-19 vaccine usage in the sporadic or clusters of local onset cases scenario.

Vaccine supply scenario	Priority groups	Vaccine usage in each substage (%)	Vaccine usage in each stage	Total vaccine usage (%)
Stage I (Very limited vaccine availability accounting for 1–10% of the city’s population)	Stage Ia: Front-line medical workers in communities with medium-high or higher onset risk and with close contacts to communities with medium-high or higher onset risk	0.61	3.67% (With 6.33% for emergency reserves)	3.67
	Stage Ib: Elderly individuals aged 80 years or above in communities with medium-high or higher onset risk and with close contacts to communities with medium-high or higher onset risk	3.06		
	Stage Ic: Emergency reserve of vaccines for utilisation in outbreak response or mitigation	6.33		
Stage II (Limited vaccine availability accounting for 11–20% of the city’s population)	Stage IIa: Front-line medical workers in communities with low to medium onset risk	0.36	10.74%	14.41
	Stage IIb: Elderly individuals aged 80 years or above in communities with low to medium onset risk	1.58		
	Stage IIc: Groups with comorbidities within communities with medium-high or higher onset risk and with close contacts to communities with medium-high or higher onset risk	0.41		
	Stage IId: Low-income groups in communities with medium-high or higher onset risk and with close contacts to communities with medium-high or higher onset risk	1.46		
	Stage IIe: Other essential workers outside the health and education sectors in communities with high onset risk and with close contacts to communities with high onset risk	6.93		
Stage III (Moderate vaccine availability accounting for 21–50% of the city’s population)	Stage IIIa: School staff in communities with medium-high or higher onset risk and with close contacts to communities with medium-high or higher onset risk	0.47	25.30%	39.71
	Stage IIIb: Remaining low-income groups in communities with low to medium onset risk	1.56		
	Stage IIIc: Remaining essential workers outside the health and education sectors in communities with low to medium-high onset risk	17.01		
	Stage IIId: Cross-district staff within communities with high onset risk and with close contacts to communities with high onset risk	6.26

**Table 3:** COVID-19 vaccine usage in the community transmission scenario.

Vaccine supply scenario	Priority groups	Vaccine usage in each substage (%)	Vaccine usage in each stage (%)	Total vaccine usage (%)
Stage I (Very limited vaccine availability accounting for 1–10% of the city’s population)	Stage Ia: Front-line medical workers	0.97	5.61	5.61
	Stage Ib: Elderly individuals aged 80 years or above	4.64		
Stage II (Limited vaccine availability accounting for 11–20% of the city’s population)	Stage IIa: Elderly individuals not covered in the first stage (aged 65 years or above)	9.33	14.21	19.82
	Stage IIb: Groups with comorbidities	2.91		
	Stage IIc: Low-income groups in communities with high onset risk and with close contacts to communities with high onset risk	1.89		
	Stage IId: Medical workers engaged in immunisation delivery	0.08		
Stage III (Moderate vaccine availability accounting for 21–50% of the city’s population)	Stage IIIa: School staff	1.19	25.96	45.78
	Stage IIIb: Remaining low-income groups with medium-high or higher onset risk and with close contacts to communities with medium-high or higher onset risk	0.83		
	Stage IIIc: Other essential workers outside the health and education sectors	23.94		

### COVID-19 vaccine usage simulations after the vaccines start to be allocated

To further evaluate the impact of the full protection generated by daily vaccination on subsequent vaccine allocation, the local onset cases in Hong Kong from 17 November 2020 to 22 December 2020 were further used to simulate the vaccine usage after the vaccines start to be allocated. During this period, the epidemic in Hong Kong was always in a community transmission scenario. In the simulation, the control measures and behavioural changes at this stage are consistent with the actual situation, and the only change is the vaccination rate. This was achieved by the value of *R*_t_ which dynamically reflects the effects of the change in vaccination rate.

According to the hypothesis (see “Methods” section), the vaccine allocations in Stage I (very limited vaccine availability accounting for 1–10% of the city’s population) started in Hong Kong on 16 November 2020. Based on the service, carrying capacity of the vaccination centres announced by the Hong Kong government, 42,000 people were able to be vaccinated each day. Thus accordingly, Stage II (limited vaccine availability accounting for 11–20% of the city’s population) could begin on 4 December 2020 at the earliest, and Stage III (moderate vaccine availability accounting for 21–50% of the city’s population) could begin on 22 December 2020 at the earliest. Moreover, the vaccines allocated for Stage I would begin to offer full protection on 14 December 2020.

**Fig. 6:**
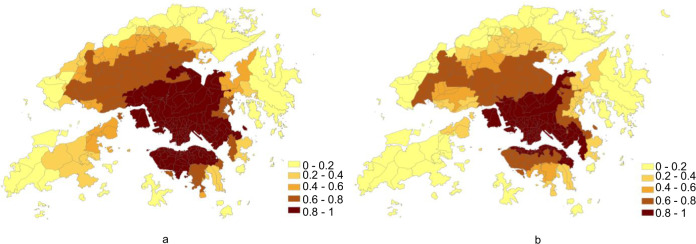
Predicted risk of COVID-19 symptom onset after vaccines starts to be allocated. **a** The accumulated results in community transmission scenario before 4 December 2020. The tertiary planning units (TPUs) with higher onset risk values are illustrated in darker colour shading. **b** indicates the accumulated results in community transmission scenario before 22 December 2020. TPUs with higher onset risk values are illustrated in darker colour shading.

Based on the above, the spatiotemporal distributions of COVID-19 onset risk from 17 November 2020 to 22 December 2020 were predicted. It would then be further combined with the accumulated predicted onset risk, regarding the community transmission scenario, before 17 November 2020. At the beginning of Stage II, the vaccine had not yet begun to provide full protection, and the spatiotemporal distribution of onset risk was affected by only external factors, such as control measures and behavioural changes. From the accumulated predicted COVID-19 onset risk in the community transmission scenario before 4 December 2020 (Fig. 6), it can be seen that the TPU ranges in the high-risk area (155 TPUs) had further expanded. Furthermore, compared to the previous prediction, with the dynamic changes in the spatiotemporal onset risk distribution (Table 3), vaccine usage in Stage II would increase by 0.24%, or 18,022 people (Table 4). However, at the beginning of the Stage III, the vaccine offered full protection within a week. The immune effect created by the vaccine gradually affected the spatiotemporal distribution of the onset risk. From the accumulated predicted onset risk in the community transmission prior to 22 December 2020 (Fig. 6), it can be seen that the area of TPUs in the high-risk area (121 TPUs) obviously shrank. Furthermore, compared to the previous prediction (Table 3), it then became apparent that vaccine usages in Stage III should, therefore cause a decrease by 20,274 people (Table 4).

**Table 4:** COVID-19 vaccine usage in the community transmission scenario after vaccines start to be allocated.

Vaccine supply scenario	Priority groups	Vaccine usage in each substage (%)	Vaccine usage in each stage (%)
Stage I (Very limited vaccine availability accounting for 1–10% of the city’s population)	Stage Ia: Front-line medical workers	0.97	5.61
	Stage Ib: Elderly individuals aged 80 years or above ≥65 years	4.64	
Stage II (Limited vaccine availability accounting for 11–20% of the city’s population)	Stage IIa: Elderly individuals not covered in the first stage (aged 65 years or above)	9.33	14.45
	Stage IIb: Groups with comorbidities	2.91	
	Stage IIc: Low-income groups in communities with high onset risk and with close contacts to communities with high onset risk	2.13	
	Stage IId: Medical workers engaged in immunisation delivery	0.08	
Stage III (Moderate vaccine availability accounting for 21–50% of the city’s population)	Stage IIIa: School staff	1.19	25.69
	Stage IIIb: Remaining low-income groups with medium-high or higher onset risk and with close contacts to communities with medium-high or higher onset risk	0.56	
	Stage IIIc: Other essential workers outside the health and education sectors	23.94	

### COVID-19 vaccine usage simulations at different real-time effective reproduction number levels

**Fig. 7:**
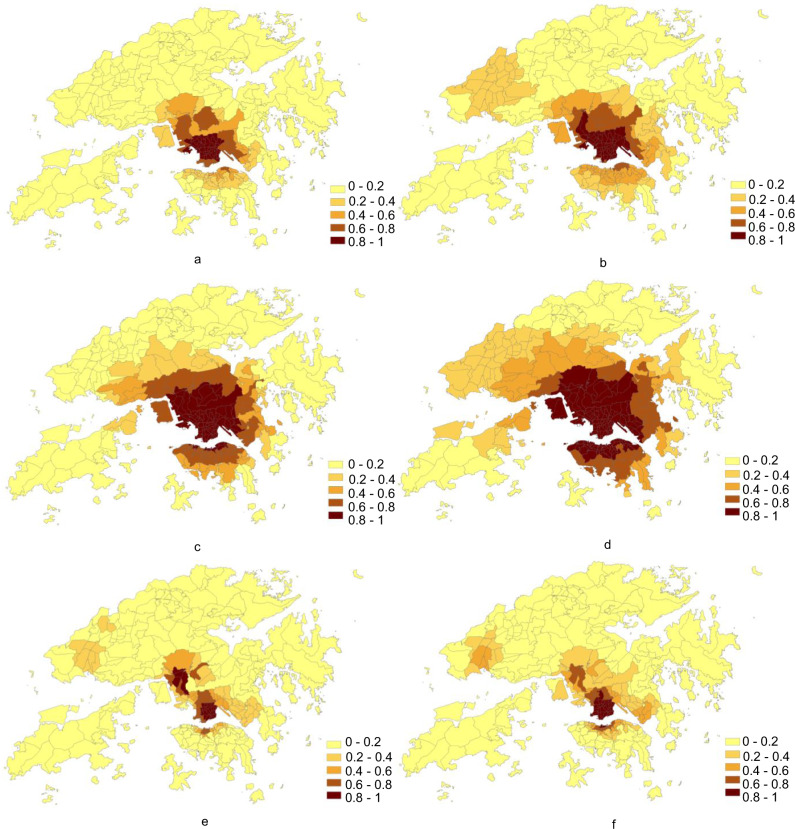
Predicted risk of COVID-19 symptom onset across Hong Kong in three epidemic setting scenarios under the impacts of different real-time effective reproductive number levels from 18 September 2020 to 16 November 2020 (a–f). **a**, **b** The accumulated results in the two scenarios of no local onset cases, and sporadic cases and clusters of local onset cases based on maintaining the current real-time effective reproduction number level, respectively. **c**, **d** The accumulated results in the two scenarios of no local onset cases, and sporadic cases and clusters of local onset cases based on increasing the current real-time effective reproduction number level, respectively. **e**, **f** The accumulated results in the two scenarios of no local onset cases, and sporadic cases and clusters of local onset cases based on decreasing the current real-time effective reproduction number level, respectively. The tertiary planning units (TPUs) with higher onset risk values are illustrated in darker colour shading.

In order to quantify the impact of control measures and behavioural changes on vaccines allocation, the COVID-19 symptom onset risk (Fig. 7) from 18 September 2020 to 16 November 2020 (social distancing measures remain consistent in Hong Kong during this period^45^) was simulated at the following three situations: (1) maintaining the current real-time effective reproduction number level, (2) decreasing the current real-time effective reproduction number level, and (3) increasing the real-time effective reproduction number level. The vaccine usage during each stage for two epidemiologic scenarios of the period—(i) no local onset cases and (ii) sporadic cases and clusters of local onset cases, was calculated accordingly.

In the no local onset cases scenario (Table 5), although total vaccine usage at the three current real-time effective reproduction number levels was the same, in Stages I and II, when increasing the existing real-time effective reproduction number level, usages were 0.53%, i.e., (39,797 persons) more, on average, than those under the condition in which the existing current real-time effective reproduction number level was maintained. Vaccine usage under the condition of decreasing the existing real-time effective reproduction number level will thus be 0.69% (51,812 persons) less than that need for maintaining the existing current real-time effective reproduction number level. In the sporadic cases and clusters of local onset cases scenario (Table 6), when the current real-time effective reproduction number level increases more greatly than that required for maintaining the existing real-time effective reproduction number level, the vaccine will then need to cover at least 7.32% of the population (549,658 persons). Conversely, if the existing real-time effective reproduction number level is further decreased, the population to be then, covered by the vaccine, can be reduced by 4.69% (352,172 persons). At the same time, at each stage, vaccine usage in the case of increasing the current real-time effective reproduction number level will be more than 1.5 times that under the situation of maintaining the current real-time effective reproduction number level. Vaccine usage under the condition of maintaining the current real-time effective reproduction number level will also be 1.5 times that needed to enable a decrease in the current real-time effective reproduction number level.

**Table 5:** COVID-19 vaccine usage in the no local onset cases scenario for situations of different real-time effective reproduction number levels.

Vaccine supply scenario	Total vaccine usage—Maintaining the current real-time effective reproduction number level	Total vaccine usage—Increasing the current real-time effective reproduction number level	Total vaccine usage—Decreasing the current real-time effective reproduction number level
Stage I (Very limited vaccine availability accounting for 1–10% of the city’s population)	0.36% (With 9.64% for essential travellers at risk + emergency reserve in Stage I)	0.64% (With 9.36% for essential travellers at risk + emergency reserve in Stage I)	0.23% (With 9.77% for essential travellers at risk + emergency reserve in Stage I)
Stage II (Limited vaccine availability accounting for 11–20% of the city’s population)	3.48% (With 6.88% for essential travellers at risk + emergency reserve in Stage II)	4.54% (With 6.10 % for remaining travellers at risk + emergency reserve in Stage II)	2.10% (With 8.13% for remaining travellers at risk + emergency reserve in Stage II)
Stage III (Moderate vaccine availability accounting for 21–50% of the city’s population)	30.86%	30.86%	30.86%

**Table 6:** COVID-19 vaccine usage in the sporadic cases or clusters of local onset cases scenario for situations of different real-time effective reproduction number levels.

Vaccine supply scenario	Total vaccine usage—Maintaining the current real-time effective reproduction number level	Total vaccine usage—Increasing the current real-time effective reproduction number level	Total vaccine usage—Decreasing the current real-time effective reproduction number level
Stage I (Very limited vaccine availability accounting for 1–10% of the city’s population)	2.47% (With 7.53% for emergency reserve)	4.15% (With 5.85% for emergency reserve)	1.14% (With 8.86% for emergency reserve)
Stage II (Limited vaccine availability accounting for 11–20% of the city’s population)	12.02%	18.14%	7.77%
Stage III (Moderate vaccine availability accounting for 21–50% of the city’s population)	39.28%	46.60%	34.59%

## Discussion

The NASEM, US, advises that geographic tools should be used to help make decisions about vaccine allocation^23^. Therefore, a spatial and dynamic solution for vaccine allocation, based on limited COVID-19 vaccine supply, has been proposed for the first time in this study. The proposed solution is in line with the principles of the WHO SAGE roadmap for prioritising COVID-19 vaccine usage in the context of this limited supply^22^.

The proposed solution is designed in the following three steps. Firstly, the accumulated risk of COVID-19 symptom onset at the urban-community scale is computed for each urban community (e.g., each of the 291 TPUs in Hong Kong) for the three epidemiologic setting scenarios: (i) no local onset cases, (ii) sporadic and cluster of local onset cases, and (iii) community transmission. The newly proposed urban-community-scale onset risk prediction model is used for this computation. The real-time effective reproduction number indicating real-time transmissibility is further included in the model as a new improvement. Secondly, vaccine usage in the three epidemic setting scenarios was computed, based on the corresponding allocation guidelines of the WHO SAGE. Thus, all available vaccine doses could be distributed to subgroups (30–46% of the total population) during each stage of each of the three scenarios. The characteristics of the BNT162b2 vaccine (one of the main vaccines allocated by the COVAX facility) and the vaccination capacity in Hong Kong have also been considered. Moreover, when the vaccination is started, the impact of the dynamic changes in the spatiotemporal distribution of the onset risk caused by influencing factors such as vaccination on the subsequent vaccine needs is simulated. Third, the impact of different real-time effective reproduction number levels on vaccine usage was also computed.

The proposed solution has the characteristics of being spatial, dynamic, and accurate, hence enriching the social vulnerability index suggested by the US NASEM for vaccine allocation.

*Spatial*. Vaccine demand is computed based on geographic location information, including (a) the spatial distribution of COVID-19 risk among urban communities, (b) risk accumulation in the corresponding community, and (c) the spatial distribution of the population subgroup in each of urban community.

*Dynamic*. Vaccine usage can be computed dynamically based on daily prediction data generated by the proposed urban-community-scale COVID-19 symptom onset risk prediction model and the corresponding accumulated risk, for each community for each of the three scenarios, prior to the latest date of disease onset. In the proposed model, the dynamic mobility of people within a community and between communities is also considered. In addition, the dynamic demand change in vaccine usage is also computed, in accordance with the change in the real-time effective reproduction number level indicating daily vaccine coverage, control measures and behavioural changes.

*Accurate*. The proposed solution to the problem of vaccine availability is at the fine-scale urban community level, which is particularly important for the situation of limited vaccine supply during the early stage. This solution enables vaccination supply to reach those groups in high-risk community areas or those subgroups with high exposure risks and mortality. It is of note that, the prediction of COVID-19 symptom onset risk is broken down at the daily level, and further that the precise prediction of the COVID-19 development trend is used for vaccine usage computation.

Hong Kong, with a population of 7,509,000^46^ and an area of 1106 square kilometres, was chosen to apply the proposed solution to demonstrate its applicability for COVID-19 vaccine allocation.

Firstly, based on the results of the daily onset risk prediction during the 296 days following the outbreak of the epidemic on 18 January 2020 in Hong Kong, the spatial distributions of accumulated onset risk in three epidemic setting scenarios were obtained through the accumulation. The COVID-19 vaccine usage in three epidemic setting scenarios before the vaccine allocation was then, computed through the proposed allocation solution. The estimated vaccine usages in the three stages of three epidemiologic setting scenarios were 30.86–45.78% of the total population in Hong Kong, or 2,317,277–3,437,620 people. The estimated usage in these three scenarios was within the total vaccine availability limited to each stage. In particular, the proposed allocation solution will protect 226,772 people and 204,245 people in low-income groups in two respective epidemiologic setting scenarios: (i) sporadic cases and clusters of local onset cases and (ii) community transmission. This example demonstrates the feasibility of the WHO SAGE roadmap for allocating limited COVID-19 vaccines for urban-level application. Moreover, this example proves that improvements to the vaccine allocation plan based on the characteristics of the spatiotemporal onset risk prediction model are reasonable.

Second, as an extension of the WHO SAGE roadmap, this solution further considers reducing the community-level spread of COVID-19. In the case of Hong Kong, the most common epidemiological setting scenario is that of sporadic cases or clusters of local onset cases. People may travel to high-risk areas for work, and this will be especially considered in Stage III of vaccine allocation computation. Such people account for 6.26% of the total population in Hong Kong. That is, 470,063 more people will need to be vaccinated due to their travel to high-risk areas. In fact, vaccinating this group of people who are travelling to higher-risk areas will help reduce the level of COVID-19 transmission between higher-risk and lower-risk areas and thus benefit overall pandemic control in the city.

Third, after the start of vaccination, with the dynamic changes in the COVID-19 onset risk under the influence of vaccination, the vaccine allocation in the subsequent stages also changes. The spatiotemporal distributions of the daily onset risk during the 35 days after 16 November, 2020 were used to simulate the dynamic changes in the vaccine allocation. Before vaccines started to offer protection, as the high-risk area expands, the needs of vaccine at the beginning of Stage II would increase by 18,022 people. Subsequently, at the beginning of Stage III, as vaccines began to offer full protection for one week, the area with high onset risk was shrinking, and the need for vaccines would decrease by 20,274 people. This simulation shows that this solution has the potential to dynamically adjust the vaccine usages of each stage according to the dynamic changes in the distribution of onset risk (including the impact of protection after vaccination).

Fourth, the vaccine demand under different real-time effective reproductive number levels indicates real-time transmissibility was also computed for Hong Kong, to quantify the impact of the control measures and behavioural changes on vaccine allocation. If the current real-time effective reproductive number level in Hong Kong is increased, then the vaccine usages needed will be increased by more than 7%, i.e., for over 549,658 more people in the scenario of sporadic and cluster of local onset cases. Conversely, for the same scenario, if the current real-time effective reproductive number level is deceased, then the vaccine usages needed will be reduced by more than 4%, i.e., for over 352,172 people. Therefore, in the case of limited vaccines, the appropriate control measures should be maintained to ensure more effective epidemic control.

This solution has the potential to help low-income countries achieve the following goals by allocating limited vaccines spatially and dynamically: (i) effectiveness: With the spatiotemporal variations of the onset risk, the allocation of vaccines can be continuously adjusted dynamically to provide limited vaccines to people who need it most, to provide the most effective protection when vaccines are limited; (ii) fairness: Vulnerable or susceptible subgroups have been considered in each stage of the vaccine allocation; (iii) reasonableness: A comprehensive assessment of the impact of vaccination, control measures, and behaviour changes on the spatiotemporal distribution of onset risk is not only conducive to adjusting vaccine distribution, but also assisting in epidemic prevention.

The proposed solution can generally apply to cities, states, and country levels. However, it must be noted that, the scope of this research mainly relates to the finer-scale city level, which is the most complex. For the state and country levels, the general logical flow of the solution is the same, just replacing the urban-community-scale WKDE model with the extended WKDE model^29^.

The authors acknowledge several limitations of this study. Firstly, the official confirmed case data in Hong Kong provides more detailed locational information than most other regions of the world, but the location of most cases were still only their residential locations. In this case, the urban-community-scale WKDE model could well represent the risk incurred by the activities of the cases in and near their residential communities, but not in and near their workplaces. If more comprehensive data on the spatial distribution of workplaces of the confirmed cases is available in future, the model prediction and the vaccine allocation plan could be more accurate. Secondly, human mobility within and between TPUs in Hong Kong was estimated based on traffic flow on Hong Kong strategic routes, which does not fully cover other traffic mode, due to traffic data accessibility in Hong Kong. Thirdly, the bandwidth selection method used in this study is a simple and alternative way to determine the bandwidth matrix. The adaptive bandwidth selection method should be further considered for further improving the bandwidth selection method^47–51^ of the urban-community-scale WKDE model. This will further enhance the application of the model in high-population-density urban areas such as Hong Kong.

Hong Kong is one of the 190 countries/regions of the COVAX Facility. The study of Hong Kong by using the proposed vaccine allocation solution can also be widely applied for other countries/regions of the COVAX Facility, which is committed to the fair allocation of the vaccine among all countries, especially for lower-income countries with minimal access to vaccination resources.

## Supplementary information

Supplementary Information

Reporting Summary

## Data Availability

Data on COVID-19 cases can be accessed at https://chp-dashboard.geodata.gov.hk/covid-19/en.html^35^. Traffic flow data can be accessed at https://data.gov.hk/en-data/dataset/hk-td-tis_2-traffic-snapshot-images^37^. Apple Mobility data can be accessed at https://covid19.apple.com/mobility^38^. Google Mobility data can be accessed at https://www.google.com/covid19/mobility/^39^. The statistics data in 291 Tertiary Planning Units (TPUs) of Hong Kong can be accessed at https://www.bycensus2016.gov.hk/en/bc-dp-tpu.html^40^. The data generated or analyzed during this study can be accessed as Data Set-vaccine allocation at 10.5281/zenodo.5121557^52^. Source data for all figures in the manuscript can be accessed as Data Set-vaccine allocation/result at 10.5281/zenodo.5121557^52^.

## Code availability

The MATLAB scripts developed and used in this study can be accessed as Onset risk-WKDE/code at 10.5281/zenodo.5121492^53^.
