# Supplementary material for: A spatial and dynamic solution for allocation of COVID-19 vaccines when supply is limited
Source: Commun Med (Lond). 2021 Aug 19;1:23. doi: 10.1038/s43856-021-00023-1 (PMC9053274; doi:10.1038/s43856-021-00023-1)
Supplement: Supplementary file 1 — Supplementary Information [file 43856_2021_23_MOESM1_ESM.pdf]

A spatial and dynamic solution for allocation of COVID-19 vaccines when supply is limited

Wenzhong Shi[1,3], Chengzhuo Tong[1,3]*, Anshu Zhang[1,3], Zhicheng Shi[2,3]

[1]Smart Cities Research Institute and Department of Land Surveying and Geo-Informatics, The Hong Kong Polytechnic University, 999077, Hong Kong, China

[2]Research Institute for Smart Cities, School of Architecture and Urban Planning, Shenzhen University, 518060, Shenzhen, China

[3]These authors contributed equally: Wenzhong Shi, Chengzhuo Tong, Anshu Zhang, Zhicheng Shi

*Correspondence author. Email: chengzhuo.tong@connect.polyu.hk

A spatial and dynamic solution for allocation of COVID-19 vaccines when supply is limited

Supplementary Table S1: 18 categories of statistics data[1] in 291 TPUs................................ 1

Supplementary Table S2: COVID-19 vaccine allocation plan[2] for the no local onset cases scenario 2

Supplementary Table S3: COVID-19 vaccine allocation plan[2] for the sporadic cases or clusters of local onset cases scenario 3

Supplementary Table S4: COVID-19 vaccine allocation plan[2] for the community transmission scenario 4

Supplementary Fig. 1: Predicted risk of COVID-19 symptom onset across Hong Kong(a-m).. 5

Supplementary References.. 6

Supplementary Table S1: 18 categories of statistics data[1] in 291 TPUs

Data Source	Subgroup
**Medical workers**	Doctors
	Nurses
**Education**	Preschool staff
	Primary school staff
	Secondary school staff
	Higher education institution staff
	Special school staff
**Elderly individuals**	Aged 65 years or above
	Aged 80 years or above
**Comorbidities**	Cardiovascular diseases
	Chronic respiratory diseases
	Diabetes
	Cancers
**Basic social service security staff**	Import/export, wholesale and retail trades
	Transportation, storage, postal and courier services
	Accommodation and food services
	Public administration, education, human health and social work activities
**Other**	People working across TPUs
	Low-income groups

Supplementary Table S2: COVID-19 vaccine allocation plan[2] for the no local onset cases scenario

Vaccine supply stage	Priority groups
**Stage I**<br>(very limited vaccine availability accounting for 1-10% of the city's population)	**Stage Ia:** Front-line medical workers in communities with high onset risk and with close contacts to communities with high onset risk<br>**Stage Ib:** Border protection staff and workers for outbreak management<br>**Stage Ic:** Essential travellers facing risk of infection outside the city<br>**Stage Id:** Emergency reserve utilization for focused outbreak response
**Stage II**<br>(limited vaccine availability accounting for 11-20% of the city's population)	**Stage IIa:** Front-line medical workers in communities with low to medium-high risk<br>**Stage IIb:** Elderly individuals defined by age-based risk (i.e., the mortality rate of each age group) with medium-high or higher onset risk and communities with close contacts to communities with medium-high or higher onset risk<br>**Stage IIc:** Remaining travellers facing risk of infection outside of Hong Kong<br>**Stage IId:** Emergency reserve of vaccine utilization for outbreak mitigation
**Stage III**<br>(moderate vaccine availability accounting for 21-50% of the city's population)	**Stage IIIa:** Elderly individuals defined by age-based risk (i.e., the mortality rate of each age group) in communities with low to medium onset risk<br>**Stage IIIb:** School staff<br>**Stage IIIc:** Other essential workers outside the health and education sectors

Supplementary Table S3: COVID-19 vaccine allocation plan[2] for the sporadic cases or clusters of local onset cases scenario

Vaccine supply scenario	Priority groups
**Stage I** (very limited vaccine availability accounting for 1-10% of the city's population)	**Stage Ia:** Front-line medical workers in communities with medium-high or higher onset risk and with close contacts to communities with medium-high or higher onset risk
	**Stage Ib:** Elderly individuals defined by age-based risk (i.e., the mortality rate of each age group) in communities with medium-high or higher onset risk and with close contacts to communities with medium-high or higher onset risk
	**Stage Ic:** Emergency reserve of vaccine for utilization for outbreak response or mitigation
**Stage II** (limited vaccine availability accounting for 11-20% of the city's population)	**Stage IIa:** Front-line medical workers in communities with low to medium onset risk
	**Stage IIb:** Elderly individuals defined by age-based risk (i.e., the mortality rate of each age group)in communities with low to medium onset risk
	**Stage IIc:** Groups with comorbidities in communities with medium-high or higher onset risk and with close contacts to communities with medium-high or higher onset risk
	**Stage IId:** Low-income groups in communities with medium-high or higher onset risk and with close contacts to communities with medium-high or higher onset risk
	**Stage IIe:** Other essential workers outside the health and education sectors in communities with high onset risk and with close contacts to communities with high onset risk
**Stage III** (moderate vaccine availability accounting for 11-20% of the city's population)	**Stage IIIa:** School staff in communities with medium-high or higher onset risk and with close contacts to communities with medium-high or higher onset risk
	**Stage IIIb:** Remaining low-income groups in communities with low to medium onset risk
	**Stage IIIc:** Remaining essential workers outside the health and education sectors in communities with low to medium-high onset risk
	**Stage IIId:** Cross-district staff in communities with high onset risk and with close contacts to communities with high onset risk

Supplementary Table S4: COVID-19 vaccine allocation plan[2] for the community transmission scenario

Vaccine supply scenario	Priority groups
**Stage I**<br>(very limited vaccine availability accounting for 1-10% of the city's population)	**Stage Ia:** Front-line medical workers<br>**Stage Ib:** Elderly individuals defined by age-based risk (i.e., the mortality rate of each age group)
**Stage II**<br>(limited vaccine availability accounting for 11-20% of the city's population)	**Stage IIa:** Elderly individuals not covered in the first stage (aged 65 years or above)<br>**Stage IIb:** Groups with comorbidities<br>**Stage IIc:** Low-income groups in communities with high onset risk and with close contacts to communities with high onset risk<br>**Stage IId:** Medical workers engaged in immunization delivery
**Stage III**<br>(moderate vaccine availability accounting for 21-50% of the city's population)	**Stage IIIa:** School staff<br>**Stage IIIb:** Remaining low-income groups with medium-high or higher onset risk and with close contacts to communities with medium-high or higher onset risk<br>**Stage IIIc:** Other essential workers outside the health and education sectors

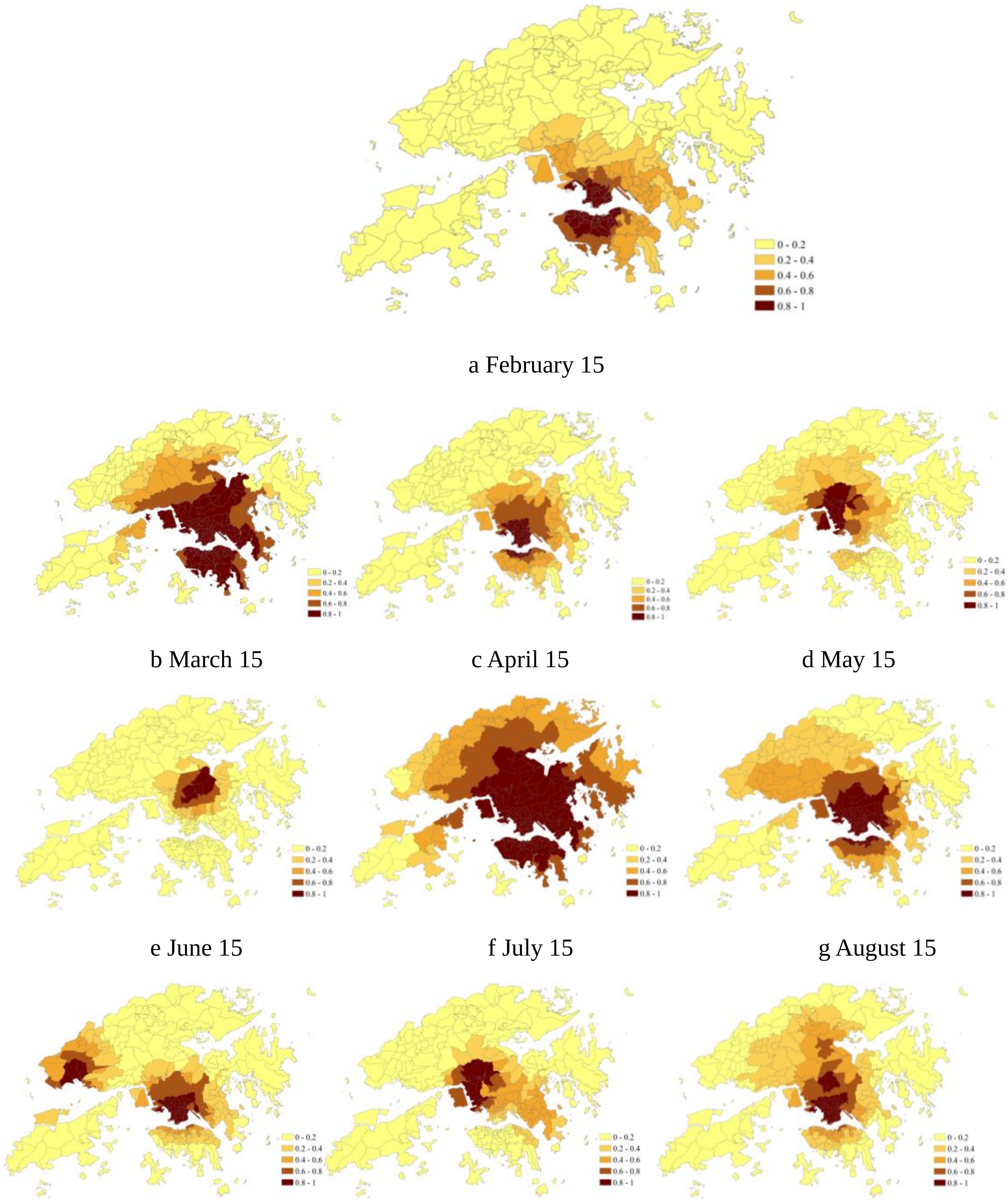

a February 15

b March 15 c April 15 d May 15

e June 15 f July 15 g August 15

h September 15 i October 15 j November 15

Supplementary Fig. 1: Predicted risk of COVID-19 symptom onset across Hong Kong(a-m). The tertiary planning units (TPUs) with higher onset risk values are illustrated in darker colour shading.

Supplementary References

1. The Census and Statistics Department of Hong Kong. *Population censuses/by-censuses.* https://www.bycensus2016.gov.hk/en/bc-dp-tpu.html (2016).

2. WHO. *WHO SAGE Roadmap for Prioritizing Uses of COVID-19 Vaccines in the Context of Limited Supply* (World Health Organization, 2020).
