# Supplementary material for: A spatial and dynamic solution for allocation of COVID-19 vaccines when supply is limited
Source: Commun Med (Lond). 2021 Aug 19;1:23. doi: 10.1038/s43856-021-00023-1 (PMC9053274; doi:10.1038/s43856-021-00023-1)
Supplement: Supplementary file 2 — Reporting Summary [file 43856_2021_23_MOESM2_ESM.pdf]

nature research

Corresponding author(s): Chengzhuo Tong

Last updated by author(s): Jul 30, 2021

Reporting Summary

Nature Research wishes to improve the reproducibility of the work that we publish. This form provides structure for consistency and transparency in reporting. For further information on Nature Research policies, see our Editorial Policies and the Editorial Policy Checklist.

Statistics

For all statistical analyses, confirm that the following items are present in the figure legend, table legend, main text, or Methods section.

n/a	Confirmed	
☐	☒	The exact sample size (*n*) for each experimental group/condition, given as a discrete number and unit of measurement
☒	☐	A statement on whether measurements were taken from distinct samples or whether the same sample was measured repeatedly
☒	☐	The statistical test(s) used AND whether they are one- or two-sided<br>*Only common tests should be described solely by name; describe more complex techniques in the Methods section.*
☒	☐	A description of all covariates tested
☒	☐	A description of any assumptions or corrections, such as tests of normality and adjustment for multiple comparisons
☐	☒	A full description of the statistical parameters including central tendency (e.g. means) or other basic estimates (e.g. regression coefficient) AND variation (e.g. standard deviation) or associated estimates of uncertainty (e.g. confidence intervals)
☒	☐	For null hypothesis testing, the test statistic (e.g. *F*, *t*, *r*) with confidence intervals, effect sizes, degrees of freedom and *P* value noted<br>*Give P values as exact values whenever suitable.*
☒	☐	For Bayesian analysis, information on the choice of priors and Markov chain Monte Carlo settings
☒	☐	For hierarchical and complex designs, identification of the appropriate level for tests and full reporting of outcomes
☒	☐	Estimates of effect sizes (e.g. Cohen's *d*, Pearson's *r*), indicating how they were calculated

Our web collection on statistics for biologists contains articles on many of the points above.

Software and code

Policy information about availability of computer code

Data collection	No software was used for data collection.
Data analysis	The MATLAB scripts developed and used in this study can be accessed as Onset risk-WKDE/code at http://doi.org/10.5281/zenodo.5121492.

For manuscripts utilizing custom algorithms or software that are central to the research but not yet described in published literature, software must be made available to editors and reviewers. We strongly encourage code deposition in a community repository (e.g. GitHub). See the Nature Research guidelines for submitting code & software for further information.

Data

Policy information about availability of data

All manuscripts must include a data availability statement. This statement should provide the following information, where applicable:

- Accession codes, unique identifiers, or web links for publicly available datasets
- A list of figures that have associated raw data
- A description of any restrictions on data availability

Data on COVID-19 cases can be accessed at https://chp-dashboard.geodata.gov.hk/covid-19/en.html. Traffic flow data can be accessed at https://data.gov.hk/en-data/dataset/hk-td-tis_2-traffic-snapshot-images. Apple Mobility data can be accessed at https://covid19.apple.com/mobility. Google Mobility data can be accessed at https://www.google.com/covid19/mobility/. The statistics data in 291 Tertiary Planning Units (TPUs) of Hong Kong can be accessed at https://www.bycensus2016.gov.hk/en/bc-dp-tpu.html. The data generated or analyzed during this study can be accessed as Data Set-vaccine allocation at http://doi.org/10.5281/zenodo.5121557. Source data for all figures in the manuscript can be accessed as Data Set-vaccine allocation/result at http://doi.org/10.5281/zenodo.5121557.

Field-specific reporting

Please select the one below that is the best fit for your research. If you are not sure, read the appropriate sections before making your selection.

☐ Life sciences ☒ Behavioural & social sciences ☐ Ecological, evolutionary & environmental sciences

For a reference copy of the document with all sections, see nature.com/documents/nr-reporting-summary-flat.pdf

Behavioural & social sciences study design

All studies must disclose on these points even when the disclosure is negative.

Study description	This is a quantitative study. First, an urban-community-scale weighted kernel density estimation (WKDE) model was proposed to predict the onset risk of COVID-19 symptoms. Secondly, illness onset risk prediction for three epidemiologic setting scenarios is developed taking into account the real-time effective reproduction numbers Rt for local cases. Finally, the vaccine demand is estimated for two different situations: a) before and or b) during the process of vaccine allocation, under the impact of daily vaccination, or control measures and behavioural changes.
Research sample	This study used all the 5,409 COVID-19 onset cases in Hong Kong with available community-level locations where they had a period of stay prior to diagnosis, reported by the Department of Health of Hong Kong. Based on the daily traffic flow statistics of 575 closed circuit televisions (CCTV) and traffic detectors covering in Hong Kong, the traffic flow data within a TPU and between TPUs are used in this research to indicate human mobility within a particular TPU and that from other TPUs to this TPU. In addition, to quantify the real-time effective reproduction number Rt for local cases in Hong Kong, community-scale daily human mobility trend data, provided by Apple Maps, were used. The other 18 categories of the statistics data in 291 TPUs, such as the number of medical workers, elderly individuals, school staff, other essential workers outside the health and education sectors, low-income groups, and immigration staff in Hong Kong, were also used in this study.
Sampling strategy	All available data for onset cases with community-level locations (N=5,409) were used for predicting the COVID-19 onset risk on future dates, without sampling.
Data collection	The data for onset cases with community-level locations, the human mobility data (including the traffic flow data and the community-scale daily human mobility trend data), and the other 18 categories of the statistics data in 291 TPUs of Hong Kong were collected manually from the public available online sources.
Timing	Data on COVID-19 onset cases in Hong Kong were collected from 18 January 2020 to 22 December 2020. To obtain the traffic flow data within a TPU and between TPUs, the daily traffic flow statistics of 575 closed circuit televisions (CCTV) and traffic detectors covering in Hong Kong were collected from 18 January 2020 to 22 December 2020, and community-scale daily human mobility trend data provided by Apple Maps and Google were collected from 18 January 2020 to 22 December 2020.
Data exclusions	Excluding the imported onset cases receiving compulsory quarantine and onset cases with unknown location information, the stated 3,316 cases were those with complete information needed by this study and were all used for forecasting future onset risks.
Non-participation	NA The data for onset cases with community-level locations, the human mobility data (including the traffic flow data and the community-scale daily human mobility trend data), and the other 18 categories of the statistics data in 291 TPUs of Hong Kong were collected from publicly available data sources, without a problem of non-participation.
Randomization	The study is not a group experiment, so the participants did not need to be allocated into groups.

Reporting for specific materials, systems and methods

We require information from authors about some types of materials, experimental systems and methods used in many studies. Here, indicate whether each material, system or method listed is relevant to your study. If you are not sure if a list item applies to your research, read the appropriate section before selecting a response.

Materials & experimental systems

n/a	Involved in the study	
☒	☐	Antibodies
☒	☐	Eukaryotic cell lines
☒	☐	Palaeontology and archaeology
☒	☐	Animals and other organisms
☒	☐	Human research participants
☒	☐	Clinical data
☒	☐	Dual use research of concern

Methods

n/a	Involved in the study	
☒	☐	ChIP-seq
☒	☐	Flow cytometry
☒	☐	MRI-based neuroimaging
